# Advances in research on the role of high carbohydrate diet in the process of inflammatory bowel disease (IBD)

**DOI:** 10.3389/fimmu.2024.1478374

**Published:** 2024-11-11

**Authors:** Ying Zhang, Linting Xun, Ran Qiao, Shumei Jin, Bing Zhang, Mei Luo, Ping Wan, Zan Zuo, Zhengji Song, Jialong Qi

**Affiliations:** ^1^ School of Medicine, Kunming University of Science and Technology, Kunming, China; ^2^ Yunnan Digestive Endoscopy Clinical Medical Center, Department of Gastroenterology, The First People’s Hospital of Yunnan Province, Kunming, China; ^3^ Colleges of Letters and Science, University of Wisconsin–Madison, Madison, WI, United States; ^4^ Yunnan Institute of Food and Drug Supervision and Control, Medical Products Administration of Yunnan Province, Kunming, China; ^5^ Yunnan Provincial Key Laboratory of Modern Information Optics, Kunming University of Science and Technology, Kunming, China; ^6^ Yunnan Clinical Research Center for Geriatric Disorders, The First People’s Hospital of Yunnan Province, Kunming, China; ^7^ Yunnan Provincial Key Laboratory of Birth Defects and Genetic Diseases, First People’s Hospital of Yunnan Province, Kunming, China

**Keywords:** inflammatory bowel disease, dietary pattern, gut microbiota, mucosa immunity, gut barrier, inflammation

## Abstract

Inflammatory bowel disease (IBD) is a chronic, systemic gastrointestinal disorder characterized by episodic inflammation that requires life-long management. Although the etiology of IBD is not fully understood, it is hypothesized to involve a multifaceted interplay among genetic susceptibility, the host immune response, and environmental factors. Previous studies have largely concluded that IBD is associated with this complex interplay; however, more recent evidence underscores the significant role of dietary habits as risk factors for the development of IBD. In this review, we review the molecular mechanisms of high-sugar and high-fat diets in the progression of IBD and specifically address the impacts of these diets on the gut microbiome, immune system regulation, and integrity of the intestinal barrier, thereby highlighting their roles in the pathogenesis and exacerbation of IBD.

## 1 Introduction

Inflammatory bowel disease (IBD), including Crohn’s disease (CD) and ulcerative colitis (UC), is a chronic condition characterized by intermittent inflammation of the gastrointestinal (GI) tract (1). The process of IBD has several detrimental effects. It results in severe GI symptoms, compromises patients’ quality of life, and imposes substantial strain on healthcare systems worldwide. Traditional studies have demonstrated that the progression of IBD is influenced by a complex interplay of genetic predispositions, environmental exposures, and immune system dysfunctions. Furthermore, recent studies suggest that dietary patterns, particularly western diets(high-sugar and high fat), are crucial elements that influence gut microbiota composition, immune regulation, and gut barrier integrity. These factors are now recognized not only as potential triggers but also as modulators of disease severity and progression, making them a critical focus for both understanding and managing IBD.

An extension study of ACCESS, encompassing eight Asian regions and Australia, reported crude incidence rates of 1.37, 0.76, and 0.54 per 100,000 persons for IBD, UC, and CD, respectively (1). Among pediatric cohorts, Northern Europe and North America have reported the highest incidences of IBD, whereas Southern Europe, Asia, and the Middle East have documented lower incidences (2). The global increase in both the incidence and prevalence of IBD, particularly among younger populations, reflects an urgent need for increased attention and intervention strategies. Although the etiology of IBD remains unclear, it affects nearly the entire intestine. Over 200 novel susceptibility loci have been identified through genome-wide association studies (GWASs), underscoring the importance of genetic risk factors in the progression of IBD (3). Among these genes, NOD2 was the first gene that has been proven to increase the risk of developing CD (4). Additionally, disruptions in genes that are involved in DNA methylation (DNMT1) (5), histone ‘writers’ (SETDB1, EZH2, ASH1L) (6–8), DNA ‘erasers’ (MBD2, TET) (9, 10), histone ‘erasers’ (HDACs 2, 3, 7) (11–13), histone ‘readers’ (UHRF1, TRIM28, SP140) (14–16), or chromatin remodelers (SMARCA4, PBRM1) (17, 18) can affect the integrity of the gut barrier and disrupt the gut microbiome and intestinal homeostasis (**Table 1**). Environmental triggers also play a pivotal role in disease stratification. Comprehensive meta-analysis results indicated that smoking, living in urban areas, and antibiotic exposure are associated with an increased risk of developing IBD (19, 20). Interestingly, AlQasrawi D et al. reported that smoking is harmful in CD patients, whereas it has a protective effect in UC patients (21). However, the molecular mechanisms involved remain unclear. Moreover, microbial dysbiosis of intestinal bacteria, along with emerging evidence for fungi and viruses, contributes to the pathogenesis of CD and UC. Therefore, the complex environmental factors of the intestine, combined with genetic susceptibility, create a conducive environment for the onset and progression of IBD.

**Table 1:** Genetic loci associated with IBD and their corresponding results.

Role	animal strain	genetic risk factors	Results	Reference
Writers	Dnmt1loxP/loxP and Dnmt3bloxP/loxP mice	Dnmt1	Dnmt1 deletion hypomethylation and genomic instability.	(5)
		Dnmt3b	Recovery of DNA methylation state and intestinal health	
	Setdb1fl/fl mice(C57BL/6)	SETDB1	SETDB1 deletion in intestinal stem cells developed spontaneous terminal ileitis and colitis	(8)
	EZH2IEC−/− and ZH2-overexpressin mice	EZH2	EZH2 reduction directly stimulates TRAF2/5 expression to enhance TNFα-induced NF-κB signaling, and promote inflammation and apoptosis in colitis.	(6)
	Ash1l-silenced mic (C57BL/6J)	ASH1L	Ash1l facilitates TGF-β-induced Treg cell polarization *in vitro* and protects mice from T cell-mediated colitis	(7)
Erasers	Mbd2−/− and CD11cΔMbd2 mice (C57BL/6)	MBD2	Mbd2 suppresses inflammation and pathology via control of innate-epithelial cell crosstalk and T cell recruitment.	(10)
	Tet2fl/fl mice (C57BL/6)	TET	Tet2-deficient mice were more susceptible to endotoxin shock and DSS-induced colitis	(9)
	Floxed Hdac1 and Hdac2, or Hdac2 mice	HDACs	Hdac1 and Hdac2 are essential IEC homeostasis regulators.	(12)
Readers	Uhrf1fl/fl and Uhrf1YP187/188AA mice	UHRF1	Uhrf1 might contribute to dynamically regulate Tnf-α expression toward maintaining intestinal immune homeostasis.	(16)
	SP140-depleted mice	SP140	the epigenetic reader SP140 as a critical regulator of macrophage function and innate immunity that enables intestinal homeostasis.	(14)

Unhealthy dietary habits have become a focal point in understanding the dynamics of IBD. The increased incidence of IBD is strongly correlated with the adoption of a Western dietary pattern. This dietary pattern not only changed nutritional intake but also reshaped gut microbiota composition. A longitudinal study in The Netherlands, involving over 125,000 adults, identified that there is a clear association between a diet high in red and processed meats and a higher risk of UC (22). In contrast, diets rich in fiber from fruits, vegetables, and whole grains have protective effects, such as reducing inflammation and lowering the incidence of IBD (23).Given the increasing global prevalence of IBD and the modifiable nature of dietary habits, it is crucial to understand how specific dietary factors influence the onset and progression of IBD. This understanding not only informs prevention strategies but also provides potential therapeutic avenues for managing existing disease.

## 2 The impact of sugar consumption on IBD risk

Sugar is among the most essential components of energy metabolism and cellular functions. Despite its importance, the WHO recommends that free sugars constitute less than 10% of daily intake, and the Institute of Medicine recommends that added sugars be limited to no more than 25%. It is well known that high sugar consumption characterizes the Western diet. Dietary sugars are mainly hexoses, including glucose, fructose, sucrose and High Fructose Corn Syrup (HFCS). These sugars are primarily absorbed in the gut as fructose and glucose. Clinical trials and epidemiological studies have linked high sugar intake, especially from sugar-sweetened beverages, to an increased risk of developing obesity, type 2 diabetes, dyslipidemia, hypertension, and cardiovascular diseases (24, 25).

IBD patients have been reported to consume more sugar and confectionary foods than healthy control participants (26). In the European Prospective Investigation into Cancer (EPIC) study, Antoine Racine et al. (27) found that high sugar and soft drink consumption combined with low vegetable intake was linked to an elevated risk of developing UC, in line with another large cohort study (28). And this association seems to be more pronounced in female patients (29).,likely due to the influence of estrogen, on the pathogenesis and clinical course of IBD in women (30).

Excess dietary fructose consumption has a pro-colitic effect that can be explained by changes in the composition, distribution, and metabolic function of resident enteric microbiota (31). High sugar increased abundance of Proteobacteria and decreased abundance of Bacteroidetes, the imbalance of microbiota to have increased pro-inflammatory properties and decreased the capacity to regulate epithelial integrity and mucosal immunity. High fructose consumption leads to liver damage through overfeeding and weight gain, but also directly acts pro-inflammatory by impairing the gut barrier and increasing intestinal translocation of bacterial endotoxin Interestingly, glucose induced less impairment of the gut barrier compared to fructose (32). Meanwhile, glucose primarily exacerbates IBD by promoting adaptive immune responses, while fructose triggers inflammation through innate immune activation and gut barrier dysfunction In addition.

### 2.1 Dietary sugar and its profound impact on gut microbiota

The human gut microbiome, which comprises approximately 100 trillion microbes, plays a role in inflammatory diseases (33). Gut microbiota constitute a dynamic ecosystem that is strongly influenced by various factors. Among these factors, diet, particularly a high-sugar diet (HSD), plays a crucial role. High-fructose corn syrup (HFCS) consumption contributes to gut microbial dysbiosis and a reduction in diversity in the mammalian intestine (**Table 2**). Khan et al. (34) reported that short-term intake of high glucose or fructose did not trigger inflammatory responses in the healthy gut but markedly altered the composition of the gut microbiome. In particular, the abundances of the mucus-degrading bacteria *Akkermansia muciniphila (A. muciniphila)* and *Bacteroides fragilis* were increased. A recent study revealed that Amuc_2109, an enzyme secreted by *A. muciniphila*, attenuates DSS-induced colitis in mice by increasing the expression of tight junction proteins (TJPs) and reducing the expression of the NLRP3 inflammasome (43). In addition, Kim S et al. (44) demonstrated that the beneficial effect of *A. muciniphila* in the intestinal tract is associated with increased levels of acetic and propionic acids in the cecal contents of mice treated with *A. muciniphila*. These findings indicate that *A. muciniphila* contributes to tissue repair in the intestinal mucosa, which involves the production of short-chain fatty acids (SCFAs). Although *A. muciniphila* is a common component of the human and murine GI tracts and has a beneficial effect on the integrity of the intestinal mucosa, its colonization can exacerbate inflammation when intestinal dysbiosis occurs (45). Montrose et al. (31) revealed that dietary fructose creates a proinflammatory environment in the intestine. It not only promotes the proliferation of *C. rodentium*, which is considered a pathogen associated with IBD but also leads to a reduction in beneficial species such as *Lactobacillus, Bifidobacterium*, and *A. muciniphila*. Moreover, Beisner et al. (46) demonstrated that HFCS reduces the number of butyrate-producing bacteria. The abundances of *Faecalibacterium* and *Ruminococcus*, which are known as representative butyrate-producing bacteria, decrease during high-fructose diet feeding in healthy adult humans (46). In addition, the abundances of *Ruminococcaceae* and *Lachnospiraceae*, the major butyrate producers, were decreased in rats fed HFCS (47). Butyrate serves to energize colonic epithelial cells, maintain intestinal integrity, and modulate immune responses (48). Laffin et al. (36) reported that there was a significant reduction in the richness of the *Lachnospiraceae* family after only two days of exposure to a HSD. This short-term dietary shift rapidly altered the gut microbial composition, depleted SCFAs, and increased susceptibility to chemically induced colitis. Furthermore, the transplantation of gut microbiota from mice fed a HSD into germ-free mice similarly increased the susceptibility of recipients to IBD (34), emphasizing the role of diet-modified microbiota in disease progression. In addition, Wang et al. (49) reported that a HSD or high-fat diet (HFD) increased the ratio of *Firmicutes* to *Bacteroidetes* in mice. This addition also led to increased levels of *Lactobacillus*, the uncultured bacteria *Erysipelotrichaceae and Olsenella*, and the uncultured *Bacteroidales* S24-7 group, as well as increased relative abundances of *Desulfovibrio, Blautia, Catenibacterium, Bacteroides, Candidatus Saccharimona*s, and *Faecalibaculum*, in feces. Zhou et al. (50) reported that high fructose intake was associated with modulation of the gut microbiome, resulting in a reduction in the relative abundance of *Clostridium* and *Clostridium scindens* at the genus and species levels, respectively. This was followed by a decrease in secondary bile acids (SBAs), particularly isoalloLCA, which affected the balance of Th17/gut regulatory T (Treg) cells and promoted intestinal inflammation.

**Table 2:** The impact of high-sugar diet and high-fat diet on the intestinal microbiota of mice.

Animal model	Intake	Microbiota	Reference
wild-type and Il10−/− mice(C57BL/6 J)	High sugar diet(10% sugar) for 1 week	*Akkermansiaceae, Bacteroidaceae, Sutterellaceae, and Marinilabiliaceae↑；* *Lactobacillaceae, Lachnospiraceae, and Rickenellaceae↓*	(34)
Two-month-old, male C57BL/6 mice	High-sucrose (12% fat, 70% CHO (primarily sucrose)	*Clostridium ↑, Bacteroidales↓*	(35)
Female wild-type mice on a 129S1/SvimJ(6-8w)	High sugar diet (HS) (50% Sucrose; Harlan Teklad AIN76A) for a period of two days	*Trichophyllaceae↓*	(36)
Male C57BL/6J mice between 8 and 12 weeks of age at the time of GL261 tumor implantation	20% dextrose water for 5 weeks before and 2 weeks after tumor cell inoculation.	*Erysipelotrichaceae, Desulfovibrionaceae, and AC160630_f strains ↑ FR888536_f and Prevotellaceae strains decreased ↓*	(37)
C57BL/6J	Mice saturated lard-based fat, saturated milkfat-based (MF) diet for 24 days	*Bacteroidetes, B. wadsworthia ↑；* *Firmicutes.↓*	(38)
C57BL/6J male mice	High-fat, 60 kcal % fat die for 9 weeks were led to obesity	*Proteobacteria, Deferribacteres, Firmicutes/Bacteroidetes and Proteobacteria/Bacteroidetes↑;* *Bacteroidetes and Tenericutes↓*	(39)
Three-week-old male and female C57BL/6 mice	Mice were fed irradiated isocaloric, isonitrogenous diets for 5 weeks. High-fat diets contained 40% energy from olive oil, corn oil or anhydrous milk fat.	*Firmicutes to Bacteroidetes↑;olive oil :Firmicutes↑( Clostridiaceae, Peptostreptococcaceae, Ruminococcaceae, and Dorea spp ).* *milk fat:Firmicutes↑* *(Erysipelotrichales and Ruminicoccus)* *Corn oil: Firmicutes ↑ (Turicibacteraceae and Coprococcus* spp.*)*	(40)
Six-week-old male C57BL/6J mice (18–22 g)	High fat diet (60% of calories as fat)	*Firmicutes to Bacteroidetes (F/B) ↑;* *Bacteroides spp, Bacteroides vulgatus, Faecalibacterium prausnitzii↓*	(41)
5-week-old male C57BL/6J mice (18–20 g)	High sugar diet(30% sucrose)+high fat diet for 10 weeks	*Prevotellaceae, Bacteroidaceae, Bacteroides, Paraprevotellaceae, Prevotella, Enterobacteriales, Enterobacteriaceae, and Gammaproteobacteria↑;* *Rikenellaceae, Alistipes, and Bacilli↓*	(42)

“↑” indicates an increase in bacterial populations, while “↓” indicates a decrease in bacterial populations.

A recent study revealed that Noni (*Morinda citrifolia* L.) fruit polysaccharide (NFP) inhibited intestinal microbiota dysbiosis in mice with IBD (51). On the one hand, NFP reduced the abundance of pathogenic bacteria. On the other hand, it enhanced beneficial bacteria, improved SCFA levels, and supported intestinal barrier repair. Additionally, NFP decreased the ratios of p-NF-κB/NF-κB, p-JNK/JNK, and p-ERK/ERK, which contributed to the inhibition of inflammatory signaling pathways (51). Sameh Saber et al. (52) reported that rosuvastatin combined with *Lactobacillus* reduced oxidized low-density lipoprotein (Ox-LDL)-induced TXNIP and attenuated the inflammatory response by inhibiting NLRP3 inflammasome assembly. Metabolites, including SCFAs (acetate, butyrate, propionate), are produced by gut bacteria. Additionally, research has shown that the levels of SCFAs, including acetate, butyrate, and propionate, are lower in the stools of patients with IBD than in those of healthy subjects (53). Gudi, R et al. (54) reported that the ingestion of β-glucans, which are nondigestible complex dietary polysaccharides commonly present in barley, increased SCFA levels and ameliorated DSS-induced colitis. This discovery suggests a potential and effective strategy for managing UC. In summary, these findings suggest that HSDs contribute to the development of IBD by inducing substantial alterations in the gut microbiome, which compromise the intestinal barrier and promote inflammation. Strategies to modify dietary sugar intake could play a critical role in managing or preventing IBD.

### 2.2 HSDs compromise intestinal barrier function and exacerbate disease severity

The intestinal barrier is responsible for preventing the ingress of microorganisms, toxins, and antigens through the intestinal wall. It also plays a crucial role in minimizing water and electrolyte loss while facilitating nutrient absorption and waste excretion. To maintain these functions, the intestinal barrier consists of physical, chemical, and biological components, including mucus; epithelial cells sealed by TJPs; immune cells; and the intestinal microbiota (55).

Mucus is the first line of defense in the intestinal barrier. Goblet cells are responsible for producing a thick layer of mucus that acts as a physical barrier, separating the microbial-rich lumen from the host tissue and immune cells. This mucus layer is predominantly composed of the mucin glycoprotein colonic mucin 2 (Muc2), which is notably downregulated in fructose-fed mice (56, 57). *A. muciniphila* has been identified as a mucus-degrading bacterium residing within the mucus layer. Khan et al. (34) reported that glucose increases the relative abundance of mucolytic bacteria and facilitates degradation of the mucus barrier. In addition, recent studies have demonstrated that a high-fructose diet results in a decrease in mucus thickness, increasing the proximity of the microbiota to intestinal epithelial cells (31, 34). Upon stimulation with microbes, epithelial cells increase the production of the antimicrobial peptides Reg3β and Reg3γ, which are primarily induced by interleukin (IL)-22. IL-22 itself is promoted by IL-23 in a cascade that enhances immune responses (58). Notably, royal jelly ameliorated symptoms of colonic cell apoptosis and decreased intestinal permeability by increasing the expression of TJPs, the number of goblet cells, and the secretion of Muc2 in mice with DSS-induced UC (59).

A crucial component of the physical intestinal barrier is the epithelium, where adjacent epithelial cells are connected by the apical junctional complex, which includes TJPs, adherens junctions (AJs) and desmosomes. The intestinal TJPs ZO-1, occludin (OCLN), claudin-1, and claudin-4 are critical for maintaining the integrity and function of the intestinal barrier. Cho YE et al. (60) reported that the levels of these proteins were significantly lower in fructose-exposed rats than in control rats. In addition, Ge Song et al. (61) demonstrated that sucrose and fructose worsen colon functions by inhibiting the expression of the TJ protein ZO-1 and increasing the level of lipopolysaccharide (LPS) in the plasma, which damages the intestinal barrier and intensifies inflammation. The colonic epithelium requires continuous renewal by crypt resident intestinal stem cells (ISCs) and transit-amplifying (TA) cells to maintain barrier integrity, especially after inflammatory damage. Burr AHP et al. (62) indicated that excess dietary sucrose can directly modulate intestinal crypt cell metabolism and inhibit ISC/TA cell regenerative proliferation, resulting in intestinal injury.

HSD can impair the gut barrier through both direct and indirect pathways. Directly, excessive sugar consumption weakens tight junctions between epithelial cells, while triggering oxidative stress, which further exacerbates gut damage. Indirectly, a high-sugar diet disrupts the balance of gut microbiota, reducing beneficial bacteria while increasing harmful ones, leading to further damage of the gut barrier. Moreover, high sugar intake triggers the release of pro-inflammatory cytokines and other metabolites, worsening mucosal inflammation and contributing to gut barrier dysfunction.

### 2.3 HSDs disrupt the gut immune balance

**Figure 1:**
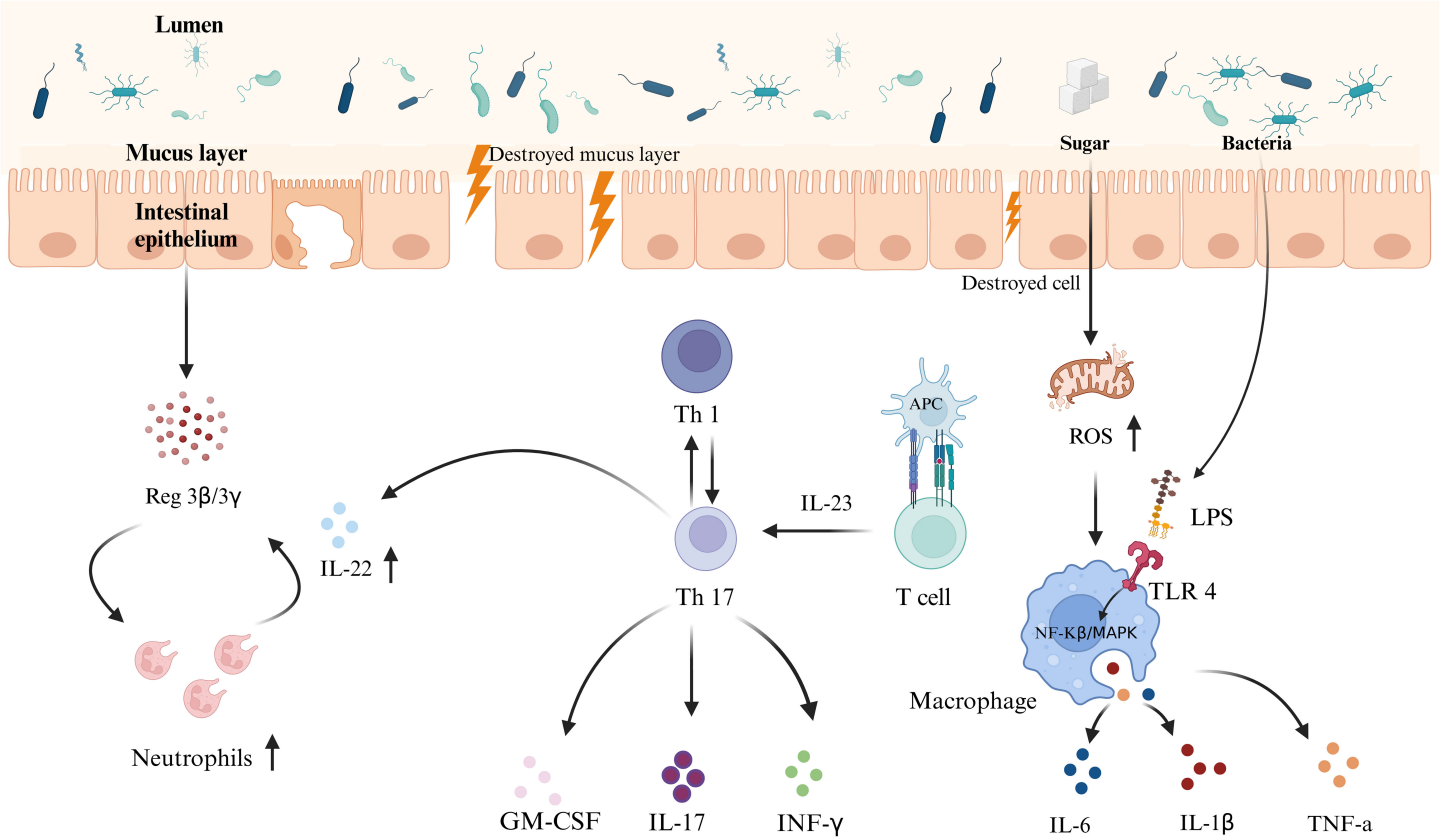
Schematic representation of the mechanism of interaction between an HSD and gut immunity. An HSD weakens the intestinal primary defense barrier by reducing Muc2 protein expression and mucus layer thickness, increasing the proximity of microbiota to the intestinal epithelium and increasing the risk of pathogen invasion. Simultaneously, an HSD enhances both adaptive and innate immune responses, including the activities of T cells, and macrophages, leading to intestinal inflammation and barrier damage. This increases the risk of developing IBD. These effects result from the combined impact of diet-induced microbiota dysbiosis and changes in immune responses.

The second line of defense consists of epithelial cells, macrophages, NK cells, monocytes, and dendritic cells (DCs). HSD was shown to increase the infiltration of these immune cells into the intestinal layers of mice (31, 63). HSDs primarily impact the adaptive immune response, amplifying the immune response mediated by T and B cells (64, 65). In contrast, a diet high in fructose predominantly influences the innate immune response, intensifying the immune response mediated by macrophages and DCs (66, 67). T lymphocytes and B lymphocytes are integral to acquired immunity. In HSD-fed mice, colitis was observed alongside elevated levels of proinflammatory cytokines, potentially influencing the T helper (Th) 1, 2, and 17 response (68). Zhang et al. (64) reported that increased glucose levels did not increase aerobic glycolysis in CD4+ T cells but instead induced increased reactive oxygen species (ROS) in the mitochondria of these cells. An increase in ROS facilitates Th17 cell differentiation via activation of the TGF-β signaling pathway. Another study revealed that glucose supported early B-cell development through glycolysis and oxidative phosphorylation (64). Activation of the mTOR/GSK3 pathway exerts an anti-B cell apoptotic effect, thereby increasing the number of B cells in the spleen and lymph nodes (65). As a primary type of mononuclear phagocyte in the intestine, macrophages play a key role in maintaining intestinal immune homeostasis (69). HFCS was shown to promote proinflammatory macrophage activation through ROS-mediated NF-κB signaling and lead to enteritis (70). Laffin et al. (36) reported that mouse bone marrow-derived macrophages (BMDMs) from HSD-fed mice released higher levels of TNF-α when stimulated with LPS than did BMDMs from chow-fed mice. Furthermore, fructose was shown to induce metabolic reprogramming in both human monocytes and mouse BMDMs, resulting in a reliance on glutamine metabolism, significantly increasing tricarboxylic acid cycle activity, and subsequently increasing the production of proinflammatory cytokines such as IL-1β, IL-6, and TNF-α in these cells (67). N. Jaiswal et al. (66) treated dendritic cells (DCs) with high fructose and then co-cultured them with T cells to investigate how fructose-induced metabolic changes in DCs affect T cell activation. They found that the exposure to high fructose promoted the formation of advanced glycation end products (AGEs) in DCs, which activated the NF-κB pathway, leading to the secretion of TNF-α by DCs. In turn, TNF-α secreted by DCs stimulated T cells to produce elevated levels of IFN-γ, thereby enhancing the inflammatory response. The impact of an HSD on the gut immunity-driven exacerbation of IBD progression is shown in **Figure 1**.

In conclusion, excessive sugar intake disrupts the intestinal barrier, enhances gut permeability, and causes profound dysbiosis of the gut microbiome. This disruption results in mucosal immune dysfunction, increasing susceptibility to infections and potentially exacerbating the development of IBD.

## 3 The impact of an HFD on IBD

An HFD has been identified as a principal factor contributing to the increasing incidence of IBD (71). Many animal experiments have demonstrated that an HFD predisposes individuals to the onset of intestinal inflammation (72, 73). In a comprehensive analysis involving 2609 IBD patients, Hou et al. (74) reported strong correlations of elevated consumption of total fats, polyunsaturated FAs (PUFAs), omega-6 FAs, and meat with an increased risk of developing UC. Multiple *in vivo* studies suggest that an HFD accelerates DSS-induced colitis by promoting obesity, activating inflammatory responses, and causing disturbances in the intestinal microbiome. These findings demonstrate the complex relationship between dietary fat intake and the pathogenesis of IBD, simultaneously emphasizing the need for further research to elucidate the subtle interactions involved in these processes.

### 3.1 HFD-driven dysbiosis as a catalyst for IBD

Research indicates that an HFD influences the composition of the gut microbiome, independent of the duration of the diet. David et al. (75) demonstrated that even a short-term HFD altered the gut microbial composition. Specifically, an HFD increases the abundance of bile-tolerant microorganisms such as *Alistipes, Bilophila*, and *Bacteroides* while decreasing the abundance of *Firmicutes*, which metabolize plant polysaccharides, including *Roseburia, Eubacterium rectale*, and *Ruminococcus bromii* (76). Additionally, Shon WJ et al. (42) demonstrated that the administration of a sucrose solution further exacerbated HFD-induced mild inflammatory condition, significantly increasing Prevotellaceae and Enterobacteriaceae levels. In mice, Prevotella*-rich* dysbiosis promoted DSS-induced colitis, leading to greater weight loss, worsened gut inflammation, and increased mortality. Jiao N et al. (77) also reported a surge in the class *Clostridia* in obese rodents. Consumption of a long-term HFD is associated with increased levels of *Bacteroides* spp.*, Bilophila wadsworthia, and Alistipes*. This finding indicates the sustained influence of dietary patterns on the gut microbiome (78). However, in the literature, the description of changes in the intestinal microbiota in an HFD environment is not entirely consistent. Some scholars believe that this inconsistency may stem from individual differences (79).

Research indicates that intake of an HFD leads to an increased prevalence of *adherent invasive Escherichia coli* (AIEC) and *Clostridioides difficile* while concurrently decreasing *A. muciniphila* and reducing populations of both *Firmicutes* and *Bacteroidetes* (80). The human microbiome is primarily composed of five bacterial phyla: *Firmicutes, Proteobacteria, Actinobacteria, Verrucomicrobia*, and *Archaea*. The most prevalent genera within these phyla are *Bacteroides, Prevotella*, and *Ruminococcus* (81). The majority of studies indicate that an HFD increases the *Firmicutes-*to-*Bacteroidetes* ratio. Westernized diets enhance the colonization of AIEC. The overgrowth of AIEC can compromise gut barrier integrity, alter host mucus production, and impair immune function in IBD. Shawki A et al. (82) reported that AIEC can increase TNF-α release, activating the TNF-α‒NF-κB regulatory pathway. This activation impacts cytoskeletal contraction and compromises intestinal permeability. In addition, the AIEC-related flagellate receptors TLR5 and NOD2 were upregulated in CEABAC10 mice fed an HFD. Increased expression of these PRRs in innate immunity has been shown to promote TNF-α synthesis and activate the inflammatory response in IBD, leading to intestinal inflammation (83). On the basis of the results of the aforementioned studies, a vicious cycle of intestinal barrier disruption has been established. A Western diet may directly increase AIEC invasion by inducing low-grade intestinal inflammation. This inflammatory state, in turn, further exacerbates AIEC invasion. Thus, the colonization of AIECs plays a crucial role in the gut inflammation induced by an HFD.

*In vivo* and *in vitro*, *A. muciniphila* has been demonstrated to increase the gene expression of TLR2, TLR4, OCLN, and claudin3, and enhances mucosal thickness, even during high-fat diet feeding, even during HFD feeding (84). Additionally, *A. muciniphila* increases anti-inflammatory Treg cells and induces antigen-specific Th cell responses in the intestines of mice. Although studies suggest that *A. muciniphila* may help protect the intestinal barrier, its role remains complex and cannot be unequivocally classified as either purely anti-inflammatory or proinflammatory. *Clostridioides difficile infection* (CDI) is known to be associated with relapse and therapeutic response in patients with IBD. The pathogenicity of *C. difficile* is attributed primarily to two major toxins: *Clostridium difficile* toxins A and B (TcdA and TcdB) and *C. difficile* transferase (CDT). Leslie J.L. et al. (85) demonstrated that toxins from CDI accelerate the degeneration of the intestinal epithelial stem cell (IESC) niche, impair IESC regeneration, and disrupt the intestinal barrier. Notably, TcdB induces epithelial cell death through a mechanism that is independent of enzymatic activity and is mediated by ROS and the NADPH oxidase complex, as demonstrated in experiments with colonic explants (86). CDI not only affects the intestinal barrier but also modulates immune responses in ASC-deficient and IL-1 receptor antagonist-treated mice. Previous studies have demonstrated that TcdA induces IL-8 synthesis in colonocytes by upregulating NF-κB and mitochondrial ROS, which leads to neutrophil chemotaxis, monocyte necrosis, and colonic inflammation (87). Moreover, Cowardin C. A et al. (88) reported that CDT decreased neutrophil recruitment by stimulating NOD-like receptors in the colon of mice. Ryan et al. (89) suggested that surface layer proteins (SLPs) from CDT may modulate DC and Th cell responses by interacting with TLR4. This research provides insights into how CDI exacerbates IBD. Recent research by Minkoff et al. (90) indicated that, compared with alternative treatments such as antibiotics, fecal microbiota transplantation (FMT) significantly enhances the resolution of recurrent CDI (rCDI) in immunocompetent adults.

The production of SCFAs is regulated mainly by gut microbiota, with Firmicutes mainly synthesizing butyrate. An HFD promotes an increase in Bacteroidetes while reducing Firmicutes, causing alterations in the gut bacterial composition characterized by this altered ratio (38, 91). Evidence has shown that mice fed an HFD have a higher *Firmicutes*-to*-Bacteroidetes* ratio than those fed a low-fat diet (LFD) (39, 40, 92). Facchin et al. (93) demonstrated that sodium butyrate supplementation enhances the growth of SCFA-producing bacteria and improves the inflammatory response in patients with IBD. Therefore, targeting SCFAs may represent a promising approach for improving the quality of life of IBD patients.

While conventional wisdom recommends that pregnant women increase nutrient intake, previous studies have revealed that an HFD during pregnancy can alter the gut microbiome of offspring and exacerbate DSS-induced colitis in adulthood (94). These findings underscore the necessity for pregnant women to maintain a balanced diet with appropriate fat intake to reduce their offspring’s risk of developing colitis.

Overall, an HFD not only alters intestinal microbiota diversity but also contributes to dysbiosis and induces intestinal inflammation (**Table 2**). In light of these results, maintaining a balanced diet with appropriate fat intake is essential for managing or preventing IBD, given the crucial role that dietary choices play in intestinal health.

### 3.2 HFDs aggravate IBD by increasing intestinal permeability

The GI tract is reinforced by a robust barrier system that protects it from toxins, bacteria, and harmful substances. This defense mechanism includes TJs, continuous renewal of epithelial layers and mucus layers, and the active involvement of the gut microbiome. Mucus derived from goblet cells plays a pivotal role in preventing bacterial breach of the enterocyte monolayer, forming a physical barrier, facilitating degradation through defensins, and initiating regulated immune responses through secreted IgA.

TJPs work in conjunction with the mucus layer and are integral to maintaining intestinal barrier integrity. These junctions consist of a network of transmembrane proteins located at the apical surface of epithelial cells, including claudins, OCLN, cingulin, TJP1, TJP2, TJ-associated MARVEL domain-containing proteins (TAMPs), and junctional adhesion molecules (JAMs) (95). In human subjects, adherence to a Western-style diet is linked to an elevated risk of developing IBD, a condition characterized by intestinal hyperpermeability (74, 96). In today’s Western diet, fat accounts for 35–45% of the total caloric intake. Chronic consumption of an HFD has adverse effects on intestinal barrier integrity. Increased intestinal permeability allows the passive translocation of bacteria, bacterial fragments, or byproducts into the lamina propria, bloodstream, and extraintestinal sites. Menta P et al. (97) reported that an HFD exacerbates intestinal permeability and upregulates the gene expression of claudin-2 while downregulating the gene expression of JAM-A and claudin-1. Kirpich IA et al. (98) examined the expression of key markers of TJ integrity and reported significant (p<0.05) downregulation of ZO-1 and claudin-1 in mice fed UFAs (corn oil/LA). Martinez-Medina M et al. (83) demonstrated that an HFD increases TNF-α secretion in CEABAC10 mice. Recent investigations have suggested that discoidin domain receptor 1 (DDR1) mediates TNF-α-induced damage to the monolayers of intestinal epithelial cells. The overexpression of DDR1 accelerates the degradation of ZO-1 and OCLN. Mechanistically, DDR1 disrupts the intestinal barrier through the NF-κB-p65-MLCK-p-MLC2 signaling pathway (99) These findings collectively affirm that dietary fat disrupts TJs and exacerbates colitis. The relationship between dietary fat and its effects on BA synthesis and gallbladder-mediated bile expulsion has been well recognized for some time. BAs are potential contributors to the development of pathogenic intestinal permeability (100). Murakami Y et al. (100) reported that HFDs increased total SBA and BA concentrations, alongside an increase in some individual BAs in the cecum. Strong positive correlations were detected between intestinal permeability and the concentrations of most SBAs, including deoxycholic acid (DCA) and ω-muricholic acid (P < 0.05). According to Wang L et al. (101), an HFD increases the percentage of gram-positive bacteria, especially those in the genus Clostridium, resulting in a notable increase in fecal DCA. The logic behind this finding is that DCA-induced macrophage polarization is mediated through the NF-κB/ERK/JNK signaling pathways downstream of TLR2. The high level of DCA induced by an HFD may trigger macrophage activation and contribute to colonic inflammation. Research has also indicated that vancomycin treatment altered the gut microbiome composition in HFD-fed mice, increasing fecal DCA levels and reducing HFD-induced colonic proinflammatory macrophage infiltration. An HFD triggers intestinal hyperpermeability via LPS turnover mechanisms (102). LPS contributes to intestinal hyperpermeability by directly modulating TJ organization, stimulating Toll-like receptor 4-cluster of differentiation 14 (TLR4-CD14)-mediated activation of NF-κB, and inducing dysfunction of intestinal epithelial cells (103).

HFDs compromise the GI barrier by altering TJ and mucus layer integrity, leading to increased intestinal permeability and inflammation. These alterations increase susceptibility to IBD by facilitating bacterial translocation and activating immune responses. Therefore, dietary modulation, such as balanced fat intake and careful diet composition, is crucial for managing gut health and preventing disease progression.

### 3.3 HFDs stimulate the immune response and accelerate the development of colitis

The long-term consumption of an HFD disrupts intestinal immune homeostasis and induces inflammation, contributing to the onset and exacerbation of IBD. Numerous epidemiological studies have linked excessive intake of HFDs with increased occurrence and relapse of IBD. HFDs and obesity are associated with intestinal low-grade inflammation (LGI), which underlies metabolic disorders and increases individuals’ susceptibility to colitis (104).

LGI is characterized by abnormal T-cell and innate lymphoid cell group 3 (ILC3) responses. Haghikia A et al. (105) demonstrated that long-CFAs (LCFAs) enhance both the differentiation and proliferation of Th1 and Th17 cells, thereby impairing intestinal barrier function through the p38-MAPK pathway. In contrast, SCFAs promote the expansion of Treg cells by suppressing the JNK1 and p38 pathways, thus supporting immune tolerance and preserving barrier integrity.

Various types of FAs exert distinct effects on intestinal immune responses. There is a close relationship between intestinal Th cells, ILC3 responses, and the microbiota profile influenced by dietary FAs. Specific colonic bacteria, such as *Helicobacter hepaticus*, can promote the differentiation of antigen-specific CD4+ T cells into Foxp3+ regulatory T cells in the colon. In contrast, segmented filamentous bacteria (SFB) drive the development of quasi-clonal proinflammatory Th17 cells in the ileum. These observations underscore the intricate interactions among diet, the microbiota, and immune responses in the pathogenesis of IBD (106–110). Furthermore, Garidou L et al. (111) proposed that the reduction in Th17 cells observed in metabolic diseases is attributed to impaired antigen-presenting cell (APC) function in the small intestinal lamina propria (SILP), which results from microbiota alterations induced by an HFD. This disruption may contribute to increased intestinal permeability, commonly referred to as a ‘leaky gut.’

CD4+CD8α+ intraepithelial lymphocytes (CD4IELs) play crucial roles in preventing abnormal inflammatory responses to self-antigens and nonpathogenic foreign antigens. The development of CD4IELs in the small intestine is dependent on microbiota. According to Bousbaine D et al., broad-spectrum antibiotics reduce CD4IELs, which subsequently rebound as the microbiota reestablishes after the cessation of antibiotic treatment (112, 113). Thus, microbiota are essential for both the differentiation and maintenance of CD4IELs. Furthermore, Cervantes-Barragan L et al. (113) reported that *Lactobacillus reuteri* metabolizes tryptophan to produce indole derivatives. The binding of these derivatives to the aryl hydrocarbon receptor (AHR) on CD4+ T cells is sufficient to downregulate the expression of the transcription factor ThPOK, thereby promoting the differentiation of CD4IELs. CD4+ T cells trigger their differentiation into CD4IELs and regulatory Tregs in mice. Bousbaine D et al. (112) identified β-hex, derived from the commensal bacterium *Parabacteroides goldsteinii*, as an antigen recognized by CD4+ T cells, which facilitates this differentiation.

HFD-induced dysbiosis is directly associated with the activation of immune cells, with a particular focus on neutrophils and macrophages in extensive research. Recent groundbreaking studies have elucidated the complex interactions between macrophages and the gut microbiome in maintaining intestinal homeostasis. In the context of IBD, the inflamed colonic mucosa attracts a substantial number of macrophages that actively secrete various cytokines, including IL-1, IL-6, TNF-α, IL-12, and IL-23. These macrophages also produce ROS, reactive nitrogen intermediates (RNIs), and proteases, all of which contribute to the degradation of the extracellular matrix (ECM). Macrophages exhibit two distinct activation states: classically activated (M1) and alternatively activated (M2). An imbalance between these M1 and M2 phenotypes has been implicated in the exacerbation of colitis in murine models of IBD (114). The regulation of macrophages in IBD involves key mechanisms such as CSF-1, the IL-10/IL-10R axis, CX3CL1-CX3CR1, and the TGF-β/TGF-βR axis, among others. Intestinal macrophages secrete a variety of cytokines to maintain tissue homeostasis (115). Wang L et al. (101) disclosed that a high level of DCA induced by an HFD may have acted as an initiator of macrophage activation and subsequent colonic inflammation.

**Figure 2:**
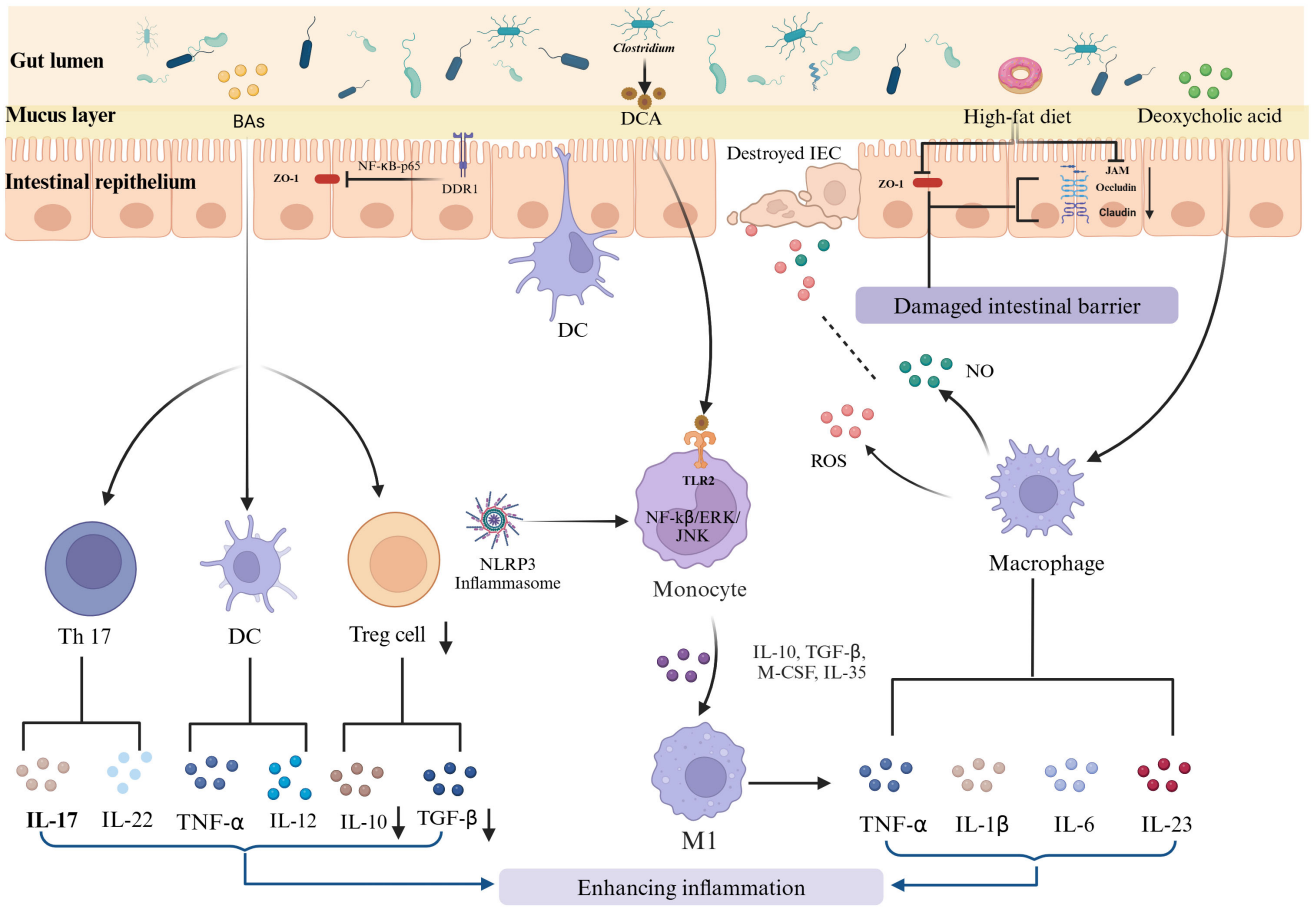
HFD-induced immune disruption and its role in IBD progression. The long-term consumption of HFD disrupts intestinal immune homeostasis, contributing to the onset and exacerbation of IBD. This disruption involves enhanced differentiation and proliferation of proinflammatory T cells, such as Th1 and Th17 cells, and a reduction in regulatory T cells. Additionally, dysbiosis induced by an HFD exacerbates the activation of macrophages. These activated macrophages produce proinflammatory cytokines and reactive species, further exacerbating the inflammatory milieu.

An HFD not only increases the proportion of gram-positive bacteria but also elevates fecal DCA levels. DCA dose-dependently promotes M1 macrophage polarization and enhances the production of proinflammatory cytokines in a dose-dependent manner, at least partially through the TLR2 pathway, which is transactivated by the M2 muscarinic acetylcholine receptor (M2-mAchR)/Src pathway. Iftikhar R et al. (116) observed that obesity induced by an HFD resulted in reduced FOXO3 levels and increased macrophage presence in the mouse colon. The activation of ERβ appears to mitigate macrophage infiltration, even in the context of HFD-induced colonic inflammation (117). Furthermore, the study by Gao T et al. (118) demonstrated that specific ablation of TBK1 in myeloid cells exacerbated inflammation in experimental colitis. Mechanistically, TBK1 functions within macrophages to inhibit the NF-κB and MAP kinase signaling pathways, thus attenuating the production of proinflammatory cytokines, particularly IL-1β. Elimination of IL-1 receptor 1 (IL-1R1) alleviated inflammatory symptoms in Tbk1-MKO mice. The role of an HFD in stimulating the immune response and accelerating colitis development is shown in **Figure 2**.

An HFD plays an important role in the development and exacerbation of IBD by disrupting intestinal immune homeostasis. This disruption manifests through the enhanced differentiation and proliferation of proinflammatory T cells, such as Th1 and Th17 cells, and a concurrent reduction in regulatory T cells. All of these changes are driven by alterations in the gut microbiome due to dietary fat intake. HFDs also cause low-grade intestinal inflammation, characterized by increased intestinal permeability (‘leaky gut’) and the activation of macrophages. Activated macrophages release a variety of proinflammatory cytokines and reactive species, which further exacerbate the inflammatory environment.

## 4 Prospects for IBD therapy

The pathogenesis of IBD remains unknown, with no definitive consensus. Investigating the interactions among genetic factors, the intestinal microbiota, environmental influences, and immune responses may offer new insights into IBD mechanisms. The specific mechanisms underlying disturbances in the balance between the immune system and the gut microbiome, including their metabolites, and the causal relationships between these factors are not yet fully understood. Moreover, examining how different dietary interventions alter microbiota composition and metabolite profiles, along with the resulting clinical outcomes, could provide valuable information for enhancing treatment strategies.

### 4.1 Gut microbiome-based therapies

The gut microbiome plays an essential role in preserving intestinal homeostasis, and emerging data reflect the promising potential of gut microbiome-based therapies for IBD. Potential therapeutic methods include probiotics, prebiotics, FMT, synthetic combinations of specific bacteria, personalized therapies on the basis of individual microbiome profiles, and BA regulation. The First International Rome Consensus Conference on Gut Microbiota and Fecal Microbiota Transplantation for Treatment advocated for FMT as a recognized treatment approach for IBD (119), although further evaluation of its long-term efficacy and safety is needed. Dysbiosis leads to an increased proportion of conjugated primary BAs in feces and a decreased proportion of SBAs due to impaired transformation in patients with IBD. Continuous exposure to SBAs has been linked to barrier inflammation and CRC (120). Therefore, BA-sensing nuclear receptors (FXR, VDR, PXR, TGR5 and VDR) are considered new therapeutic targets for treating IBD. Recent studies emphasized that gut microbiota-derived metabolites, including urolithins and tryptophan metabolites, function as critical chemo-immune regulators, facilitating host–immune interactions and enhancing tissue homeostasis and gut barrier integrity (121, 122). Although still in the early stages, emerging interventional studies that focus on dietary modulation or microbiome targeting are promising and are anticipated to play a crucial role in managing IBD. Diets such as the CD exclusion diet, the specific carbohydrate diet, and the Mediterranean diet have demonstrated efficacy in inducing clinical remission in CD patients. Moreover, dietary approaches characterized by reduced animal protein intake and increased fiber consumption have been effective in both inducing and maintaining remission in patients with UC and CD (123).

The gut microbiome presents promising potential for the treatment of IBD. Future therapeutic strategies may increasingly focus on personalizing microbiome interventions to increase treatment efficacy and safety. Monitoring and adjusting the gut microbiome at an early stage could facilitate the prevention of disease progression and provide a strategy for long-term management to mitigate the risk of relapse. In addition, integrating microbiome-based therapies with existing treatment modalities may enhance therapeutic outcomes and reduce adverse effects. Such approaches could also pave the way for the development of novel, targeted pharmacological agents.

### 4.2 Biomaterial-based interventions

Biomaterial-based bioengineering approaches and gut microbiota-derived metabolites are emerging as promising therapeutic strategies for IBD. These strategies target inflammation, intestinal permeability, and mucosal repair, thereby improving tissue homeostasis and enriching the gut microbiome. Hyaluronan (HA), a glycosaminoglycan found in synovial fluid and the ECM of various tissues, including the GI mucosa, exhibits immunomodulatory and matrix-remodeling properties (124). Lee, Y et al. (125) demonstrated the efficacy of an HA–bilirubin nanomedicine (HABN) system in modulating the gut microbiome and regulating innate immune responses in a preclinical colitis model. The HABN system was shown to reduce intestinal permeability by increasing the mRNA expression of the TJPs ZO-1 and OCLN-1. In a recent study, investigators used a biomaterial-based delivery system featuring the genetically modified probiotic *Escherichia coli* Nissle 1917 (ECN), which overexpresses catalase and superoxide dismutase (ECN-pE) to mitigate intestinal inflammation (126). Moreover, advanced clinical strategies are exploring ECM-mimicking polysaccharide biomaterials such as HA, chitosan, alginate, collagen, and pectins, which contribute to mucosal protection and repair while supporting a favorable gut microbiome. Recent findings also highlight the role of mucus PCs in restoring mucosal integrity and enhancing barrier functions (127). However, many questions and knowledge gaps must be resolved before these approaches can be clinically translated. Key areas for further investigation include the distinct mechanistic roles of biomaterials and phospholipids, their regenerative potential, their immunogenicity, and the underlying signaling mechanisms involved. Additionally, the source and purity of these materials require thorough evaluation.

Nanobiomaterials hold great promise for the treatment of IBD. They facilitate targeted drug delivery, enhance drug stability and controlled release, and minimize side effects. Zhou J et al. (128) developed a drug-free, biodegradable nanomedicine (MON-PEI) that exhibited high cfDNA binding affinity and ROS-responsive degradation, allowing for a reduced dosing frequency and effective amelioration of colitis, even with delayed treatment. However, Tan M et al. reported that IBD alters the *in vivo* distribution of orally administered nanoparticles (129). Recent advancements in extracellular vesicle (EV)-based nanotechnology have provided unprecedented opportunities for nanomedicine platforms (130).

Nevertheless, several challenges remain in the use of nanoparticles for the treatment of colitis. These challenges include enhancing enzyme-like activity or catalytic capacity, assessing long-term toxicity, further elucidating therapeutic mechanisms, and exploring potential effects on brain-related complications such as depression or anxiety.

### 4.3 Emerging drug therapies

Novel therapeutic agents for IBD are being developed (131), including Janus kinase (JAK) inhibitors that target the JAK1, JAK2, and JAK3 signaling pathways. These inhibitors aim to block various cytokine pathways implicated in IBD. In addition, integrin receptor antagonists, such as α4β7, may reduce the gut-selective trafficking of immune cells by obstructing integrins expressed on endothelial cells. Other emerging therapeutic approaches involve anti-IL agents, leukocyte trafficking inhibitors, and gut microbial metabolites that target the AhR/Nrf2 and NF-κB pathways.

The future of drug development for IBD faces several challenges. These challenges include elucidating the complex etiology of the disease, enabling personalized treatment approaches, overcoming drug resistance, ensuring long-term safety, achieving precise drug delivery, modulating the gut microbiome and addressing neurological complications associated with IBD. To address these challenges, a comprehensive understanding of the underlying pathological mechanisms is essential for developing more effective and safer therapeutic options.

## 5 Conclusion

Overall, both HSD and HFD exacerbate IBD through mechanisms involving dysbiosis, altered immune responses, and impaired intestinal barrier function. HSD predominantly increases the proliferation of pathogenic bacteria and promotes the production of inflammatory cytokines, thereby increasing immune activity and disrupting the barrier. In contrast, an HFD alters the gut microbiome by increasing bile-resistant and fat-metabolizing bacteria, induces chronic inflammation by activating immune cells, and causes barrier dysfunction by altering BA concentrations and FA toxicity. These findings not only highlight the need for targeted nutritional interventions to benefit IBD patients but also emphasize the importance of personalized dietary recommendations on the basis of individual gut microbiota profiles and dietary triggers. Integrating nutritional management with conventional medical treatments may provide a more comprehensive approach to IBD management and improve patient outcomes. Future treatment directions may include gut microbiome-based therapies, biomaterial-based interventions and emerging drug therapies that aim to improve treatment efficacy and patient quality of life through more precise and personalized approaches.
